# Structural, Impedance, and EDLC Characteristics of Proton Conducting Chitosan-Based Polymer Blend Electrolytes with High Electrochemical Stability

**DOI:** 10.3390/molecules24193508

**Published:** 2019-09-27

**Authors:** Shujahadeen B. Aziz, Rebar T. Abdulwahid, Muhamad H. Hamsan, Mohamad A. Brza, Ranjdar M. Abdullah, Mohd F. Z. Kadir, Saifful K. Muzakir

**Affiliations:** 1 Advanced Polymeric Materials Research Lab., Department of Physics, College of Science, University of Sulaimani, Qlyasan Street, Kurdistan Regional Government, Sulaimani 46001, Iraq; rebar.abdulwahid@univsul.edu.iq (R.T.A.); mohamad.brza@gmail.com (M.A.B.); ranjdar.abdullah@univsul.edu.iq (R.M.A.); 2 Komar Research Center (KRC), Komar University of Science and Technology, Kurdistan Regional Government, Sulaimani 46001, Iraq; 3 Department of Physics, College of Education, University of Sulaimani, Old Campus, Kurdistan Regional Government, Sulaimani 46001, Iraq; 4 Institute for Advanced Studies, University of Malaya, Kuala Lumpur 50603, Gombak, Malaysia; hafizhamsan93@gmail.com; 5 Manufacturing and Materials Engineering Department, Faculty of Engineering, International Islamic University of Malaysia, Kuala Lumpur 50603, Gombak, Malaysia; 6 Centre for Foundation Studies in Science, University of Malaya, Kuala Lumpur 50603, Gombak, Malaysia; mfzkadir@um.edu.my; 7 Material Technology Program, Faculty of Industrial Sciences & Technology, Universiti Malaysia Pahang, Lebuhraya Tun Razak, Gambang, Kuantan 43600, Pahang, Malaysia; saifful@ump.edu.my

**Keywords:** polymer blend electrolytes, FTIR study, impedance study, TNM and LSV analysis, EDLC fabrication, cyclic voltammetry

## Abstract

In this report, a facile solution casting technique was used to fabricate polymer blend electrolytes of chitosan (CS):poly (ethylene oxide) (PEO):NH4SCN with high electrochemical stability (2.43V). Fourier transform infrared (FTIR) spectroscopy was used to investigate the polymer electrolyte formation. For the electrochemical property analysis, cyclic voltammetry (CV), linear sweep voltammetry (LSV), and electrochemical impedance spectroscopy (EIS) techniques were carried out. Referring to the FTIR spectra, a complex formation between the added salt and CS:PEO was deduced by considering the decreasing and shifting of FTIR bands intensity in terms of functional groups. The CS:PEO:NH_4_SCN electrolyte was found to be electrochemically stable as the applied voltage linearly swept up to 2.43V. The cyclic voltammogram has presented a wide potential window without showing any sign of redox peaks on the electrode surface. The proved mechanisms of charge storage in these fabricated systems were found to be double layer charging. The EIS analysis showed the existence of bulk resistance, wherein the semicircle diameter decreased with increasing salt concentration. The calculated maximum DC conductivity value was observed to be 2.11 × 10^−4^ S/cm for CS:PEO incorporated with 40 wt% of NH_4_SCN salt. The charged species in CS:PEO:NH_4_SCN electrolytes were considered to be predominantly ionic in nature. This was verified from transference number analysis (TNM), in which ion and electron transference numbers were found to be *t_ion_* = 0.954 and *t_el_* = 0.045, respectively. The results obtained for both ion transference number and DC conductivity implied the possibility of fabricating electrolytes for electrochemical double layer capacitor (EDLC) device application. The specific capacitance of the fabricated EDLC was obtained from the area under the curve of the CV plot.

## 1. Introduction

A deep understanding of polymer electrolytes is vital for researchers, because they are useful in highly specialized interdisciplinary fields, such as electrochemistry, polymer science, organic chemistry, and inorganic chemistry [1]. Dry solid polymer electrolytes (SPEs) have captured the attention and interest of many research groups as safer alternatives to liquid electrolytes [2]. The advantages of dry solid polymer electrolytes are reflected in their reasonable mechanical strength, straightforward thin film fabrication with desirable sizes, and electrode/electrolyte contact [3,4,5,6]. SPEs are required to exhibit higher thermal and electrochemical stabilities [7]. The preparation of SPEs comprises dissolving salts in a polymeric matrix where salt provides cations and anions. Consequently, the polymers are conductive ionically and thereby usable widely in a wide range of electrochemical devices, for instance fuel cells, batteries, and capacitors [8]. To scale up to the industrial level, the polymer electrolytes need to be cheap, ionically highly conductive, and dimensionally and mechanically stable. All these are challenges that are associated with these interesting polymer electrolytes. However, apparently, the polymer electrolytes have shown to have relatively low ionic conductivity and high crystallinity [9]. As it is known, polymer electrolytes consist of two main structures, crystalline and amorphous phases. It is confirmed that amorphous region is mainly responsible for the ion transport in polymer electrolytes. To strength this structure, semi-crystalline polymers are often used as host materials in polymer electrolytes [10]. Therefore, the growing need for sufficient conductivity has encouraged researchers to consider a reasonable method, which comprises polymer blending [11,12].

Natural polymers have attracted substantial interest recently due to their unique chemical and physics properties, making them possible to be used widely as matrices for SPEs in electrochemical devices. In particular, they have been used in applications of electrochromic devices, high energy density batteries, sensors, and fuel cells [13]. Natural polymers have significant potential as alternative materials for synthetic polymer counterparts. Biodegradability, non-toxicity, and cost effective are among the most important properties of some natural polymers, such as starch, cellulose, chitosan, carrageenan, and agarose, which made them to be intensively examined [14]. Herein, among these polymers, chitosan (CS) is the most popular one. CS as deacetylated product, i.e., one of derivatives of chitin, is abundant in nature [15]. It is mainly obtainable from shrimp wastes and used in a number of applications [16]. It is reported that the enrichment of CSchains with the polar groups (NH_2_ and OH), which serve as conjunction sites with high affinity for transition metal ions, make CS a common sorbent [17]. Moreover, another impressive characteristic of CS is the ability to mold into different forms ranging from hydrogels and porous scaffolds to films [18]. To make a polymer with dominated structure, a 70 wt% CS and 30 wt% PEO produce an optimum ratio [19] and thereby the same ratio was chosen for this work with various concentration of ammonium thiocyanate(NH_4_SCN). Therefore, a reasonable polymer electrolyte with a relatively high conductivity can be obtained as electrode separators in electrochemical double layer capacitor (EDLC), which can be used to some extent as an alternative for conventional batteries. It is well-known that, based on the charge storing existence, there are two electrochemical energy storage devices; batteries and electrochemical capacitors [20]. In the former, there is an electron transfer at the interfacial region between the electrode surface and the ions in the electrolyte region, which is called Faradaic process. In the latter, there is no charge transfer, i.e., no electron transfer but instead there is a charge accumulation occurrence. This process is known as non-Faradaic process. In EDLC devices, the energy storage mechanism is based on the non-Faradaic process, where ions make a doublelayer at the interfacial region [21]. There are several advantages that make EDLC devices a viable option over supercapacitors (or pseudocapacitors), such as the relatively higher power density, durability, better thermal stability, higher reversibility, cheap cost, safety, and straightforward fabrication methodology of EDLC devices [22,23,24]. Over the last 20 years, many research groups invested efforts into the invention of polymer electrolytes (PEs) with plausible conductivity and electrochemical stability [25]. PEs are characterized by a safe behavior and promising choice to assemble as a separator in avoiding short circuits between anode and cathode during operation [26]. Recent studies discovered that ammonium salts are good proton donors [27,28,29,30]. Ammonium thiocyanate (NH_4_SCN) has low lattice energy of 605 kJ/mol compared to other ammonium salts, such as NH_4_NO_3_ (646 kJ/mol) and NH_4_I (634 kJ/mol), that can easily be dissociated into cation and anion once dissolved in the host polar polymers. Therefore, NH_4_SCN provides more ammonium ions to the polymer matrix [28,29]. According to a study reported by Srivastava et al. [30], in which a coulombmetric study on a PEO–NH_4_SCN system was carried out, H^+^ is considered to be the only contributed cation to the ionic transport. In a polymer ammonium salt system, the conducting species is H^+^ ion, which originates from one of the loosely bound protons from the ammonium ion, as already reported in the literature [31,32].Despite the fact that various energy storage systems such as lithium ion batteries, lead acid batteries, etc., are accessible; the development of new low-cost, safe, and environmentally friendly electrochemical devices is required. Proton-based devices (EDLC and batteries) may be considered a good alternative, owing to the small ionic radii of H+ ions. Furthermore, the cost-effectiveness of the electrode and electrolyte materials, as well as no associated safety issues, are the key advantages that make the proton-based devices more attractive for applications, and thus stimulate further fundamental research. On the basis of this review, the current work aimed to investigate the suitability of CS:PEO based polymer electrolyte for EDLC applications.

## 2. Results and Discussion

### 2.1. FTIR Analysis

**Figure 1:**
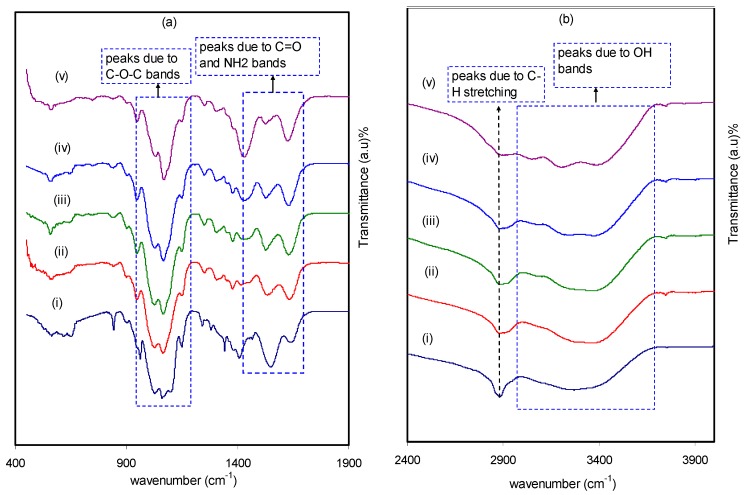
Spectrum of (i) CSPX0 (pure CS:PEO), (ii) CSPX1, (iii) CSPX2, (iv) CSPX3, and (v) CSPX4 in the range (**a**) 400 cm^−1^ to 1900 cm^−1^, and (**b**) 2400 cm^−1^ to 3900 cm^−1^.

The formation of polymeric blends has been extensively confirmed by Fourier transform infrared (FTIR) spectroscopy. The principle of this technique is based on providing information on the functional groups and intermolecular interaction. This can be ensured via the analysis of FTIR spectra, which correspond to stretching or bending vibrations of particular bonds [33]. The analysis of the obtained spectra contains observation of the shifting and change in intensity of the functional groups bands occurring in pure CS:PEO and CS:PEO:NH4SCN electrolyte samples. The FTIR spectra for the pure CS:PEO and CS:PEO:NH_4_SCN complexes at different regions of IR radiations are shown in Figure 1a,b. It should be noted that, to show a desired polymer host formation, it is required to obtain the existence of heteroatoms (e.g., O and N) with lone pair electrons [34]. From the results obtained in this study, a strong peak was centered at around 2900 cm^−1^, which is correlated to the C-H stretching modes [35,36,37]. However, it can be noted that the peak intensity is declined with increasing salt concentration. Another observation from the literature is that polymer chains containing electronegative atoms (i.e., O or N) in the repeating units could behave as solvents for particular salts [38]. This is evidenced by the attractive interaction occurring between the cations and the chains. Moreover, it was shown that CS polymer is characterized by a single NH_2_ group and a pair of OH groups in each repeating unit [10,39]. In a previous report [35], observations of a broad peak at around 3559 cm^−1^ and a strong peak at 2900 cm^−1^ have been recorded. These peaks were attributed to -OH stretching and C-H stretching modes of PEO, respectively, which indicate a high influence of material doping. It can be seen from Figure 1b that a peak at around 2904 cm^−1^, as a result of C-H stretching, is broadened as the concentration of NH_4_SCN salt increases and almost disappears at 40 wt%. The peak at around 1100–1200 cm^−1^ can be associated to C-O-C stretching mode [35,40]. As evidence, both intensity changing and peak shifting provide strong information about the complex formation between the dopant salt and CS:PEO. Each sample is characterized by the main features of absorption peaks, for example vibration of amino group (NH_2_), O=C-NHR, and hydroxyl (OH) functional groups of CS. From Figure 1a, it is seen that shifts occur towards the lower wave numbers in the bands of amino (NH_2_), O=C-NHR, and (OH) groups. This provides more insight into the formation of complexation between the NH_4_SCN salt and CS:PEO [41,42]. Another interesting observation is the change of relative band intensity (i.e., decrease, broadening, and shifting), indicating the occurrence of electrostatic interaction between the functional groups of the CS:PEO polymer blends and the ions from the salt [43]. More insights can be obtained from the shift of vibrational bands in the direction of lower wave numbers, suggesting an increase in the weakness of intermolecular and intramolecular hydrogen bonds between polymer chains. Consequently, an amorphous phase characteristic is a possible result. It is apparent that cation ions are attracted to both nitrogen and oxygen atoms at the polymer chains, resulting in lowering vibration intensity of the N–H or O=C–NHR bonds and thus a higher molecular mass is resulted [10].

### 2.2. Impedance Study

The use of SPEsin electrochemical devices has become important. In solid-state electrochemistry, a large number of research groups are devoted to develop high ion-conducting materials for applications in both the conversion and storage of energy [44]. In electrochemical impedance spectroscopy (EIS), the result is a plot between the imaginary part and the real part of the impedance. One can extract the expected model of equivalent circuit from this plot [45]. In the case of applying electric field to a system, the cations can transport from one coordinated site to another, owing to the interruption of weakly coordinated cation to the sites along the polymer chain. In order to understand the chemistry and ionic transport property of these technologically impressive materials, more efforts are required in this field [46].

**Figure 2:**
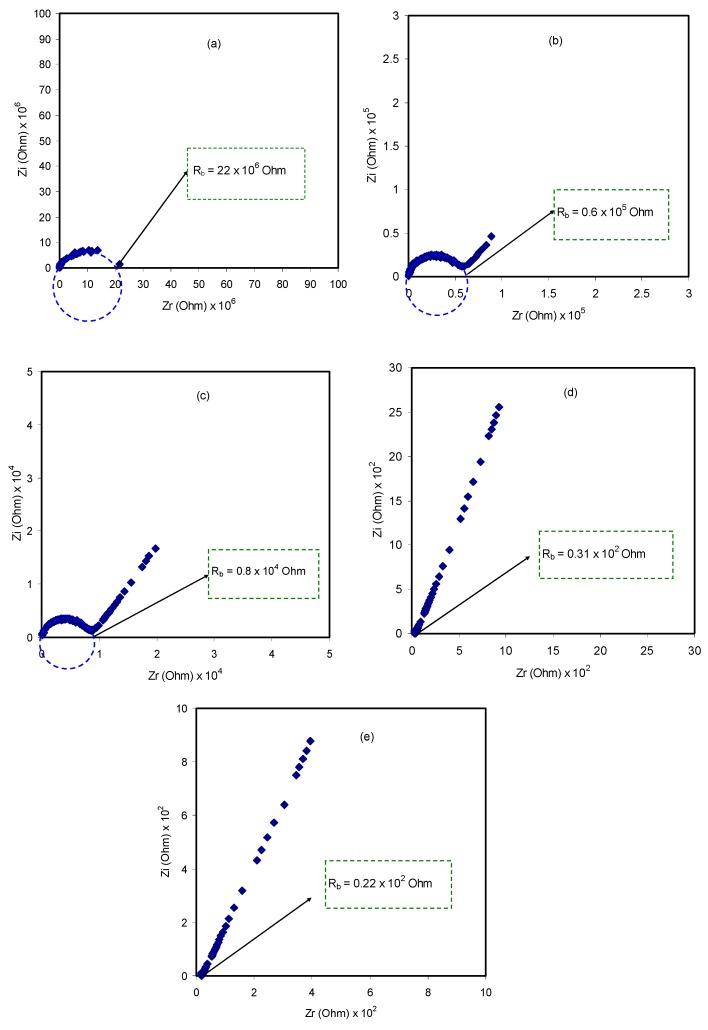
Impedance plots at ambient temperature for (**a**) Pure CS:PEO (CSPX0);(**b**) CSPX1, (**c**) CSPX2, (**d**) CSPX3, and(**e**) CSPX4 samples.

**Figure 3:**
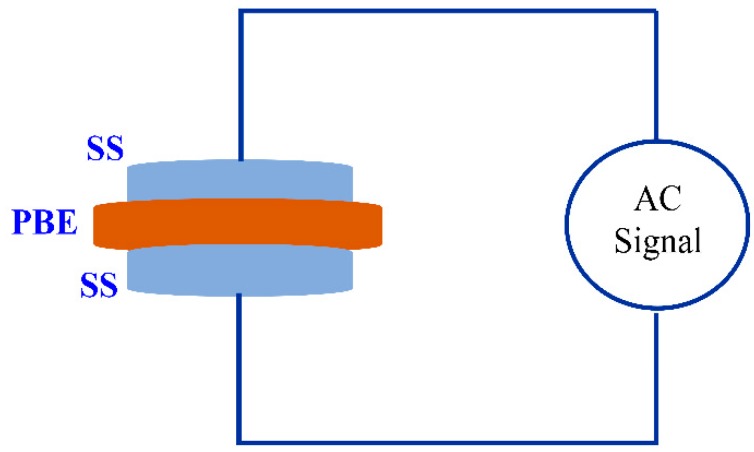
Schematic diagram of impedance measurement which consists of two identical stainless steel (SS) electrodes. The inserted sample is a polymer blend electrolyte (PBE).

The impedance plot of the blended electrolyte samples at room temperature is presented in Figure 2. At the high frequency region, it can be clearly seen that there is an incomplete semicircle, whereas at the low frequency region, there is a spike shape appearance. The former appearance is attributed to the bulk responses, i.e., electrolyte, and the latter case is attributed to the capacitor formed at the double layer in the electrode/electrolyte interface region, owing to accumulation of free charges [47,48,49]. The diameter of the semicircular portion is reduced as the salt content increases. This implies that charge carriers, which are the ions, can move throughout the sample, considering the sample body as a resistor parallel to a constant phase element in the equivalent circuit model [49]. At low frequencies, the impedance plot response is expected to be a straight line, parallel to the imaginary axis. However, double layer capacitance causes the curvature as a consequence of the electrode polarization effect at the blocking electrodes [50,51,52]. To determine the DC conductivities (σ_dc_), complex impedance (Z*) comprising the imaginary (Z_i_) and real (Z_r_) parts can be employed using the following relationship:

(1) $$\sigma_{dc}=\left(\frac{1}{R_{b}}\right)\times \left(\frac{t}{A}\right)$$

where *t*, *A*, and *R_b_* represent the thickness, area, and bulk resistance of the film, respectively. All these can be obtained from the intercept of the impedance plot on the real axis. The schematic diagram for impedance measurement is shown in Figure 3. Table 1 shows the DC conductivity values of pure CS:PEO and CS:PEO:NH_4_SCN electrolyte systems at room temperature. An obvious increase in the DC conductivity from 6.87 × 10^−10^ S/cm for pure CS: PEO to 2.1 × 10^−4^ S/cm for CS:PEO incorporated with 40 wt% of NH_4_SCN can be seen. Several research data results revealed that the polymer electrolytes of relatively high DC conductivity ranging from 10^−5^ to 10^−3^ S/cm are crucial for electrochemical device applications, including both batteries and electrical double layer capacitors (EDLCs).

### 2.3. Electrochemical EDLC Study

#### 2.3.1. TNM Analysis

**Figure 4:**
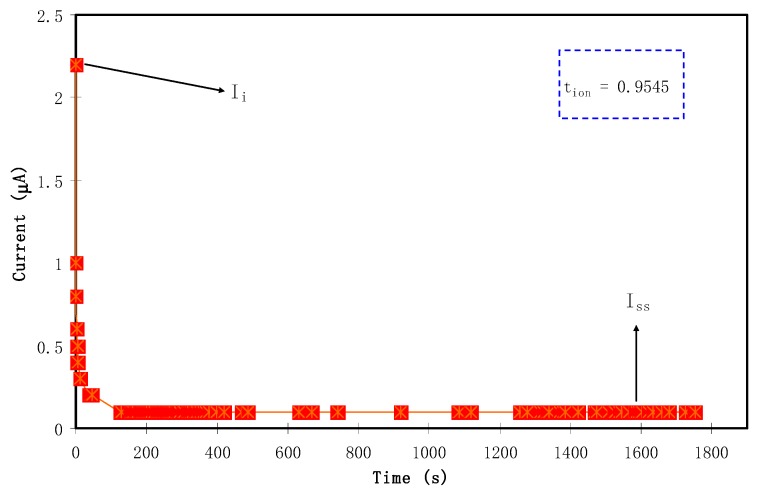
Current with respect to time plot for theCSPX4 blend electrolyte film.

To determine the main or dominant charge carrier species in polymer electrolytes, it is the best choice to use transference number measurement (TNM) using DC polarization technique. As mentioned in Section 2.3, the measurement technique was comprised of applying DC voltage to the samples with a characteristic potential window, and followed by plotting the resulting current versus time [53]. Figure 4 shows the current with respect to time plot for the CSPX4 blend electrolyte film with highest conductivity. It should be noted that the plot reveals the formation of the relatively highest conducting electrolyte, where the polarization curve shows a recording of the current that raised from 0.8 V until reaching saturation state. Accordingly, the current decayed drastically prior to reaching the steady state. At the steady state, when the polarization of the electrochemical cell is taking place, the remaining current flow can be a reflection of electron transfer rather than ion transport. This is also explained in terms of the blockage, resulting from ion accumulation at the interfacial region of stainless steel electrode/electrolyte, where only electrons can transfer between the electrolyte and solid electrode phases [54]. The relatively high conductive electrolyte connected the two stainless steel electrodes (SS) can perform facilitating charge transport and electron transfer at the interfacial region. The following equations were used to calculate both the ion (*t_ion_*) transport and the electron (*t_el_*) transference number:

(2) $$t_{ion}=\;\frac{I_{i}-I_{ss}}{I_{i}}$$

(3) $$t_{ion}=1-\;t_{el}$$

where *I_ss_* is the steady-state current and *I_i_* is the initial current. The large value of *I_i_* observed at 2.2 μA is a result of the contribution of both ion and electron at the initial stage. This phenomenon reveals the behavior of an ionic conductor that accompanies electron transfer [55]. The obtained transfer numbers *t_ion_* = 0.954 and *t_el_* = 0.045 indicate the contribution of ions as dominant charge carriers within the polymer electrolyte. The proximity of *t_ion_* to 1—the ideal value—is a worthy result, confirming the nature of the conduction mechanism of the prepared electrolyte films by ions [53]. This is caused by the possibility of Li^+^ cations’ departure from the coordinating sites of polymer chains of PEO polymer; thereby, charge transport within the polymer blend is mainly due to cationic motion only [56]. The data results obtained in the current report are higher than those documented for the carboxylmethylcellulose-NH_4_Fsystem [57]. Ultimately, it seems that in the chitosan-dextran-NH_4_F system, ions are mainly responsible for charge carrying.

#### 2.3.2. LSV Study

**Figure 5:**
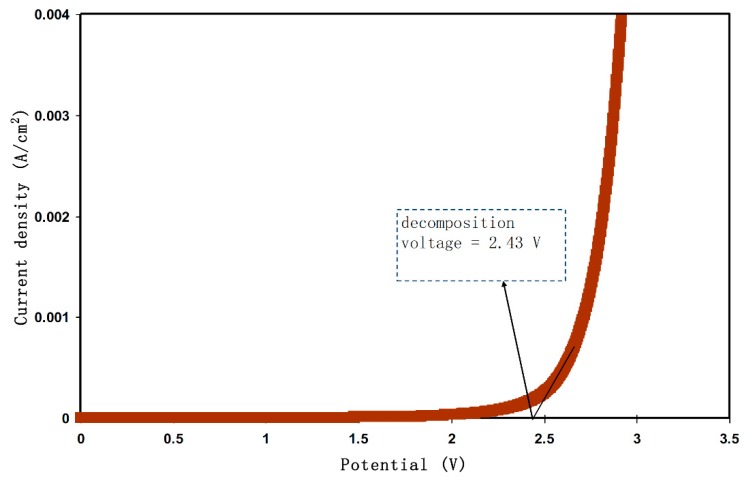
LSV curve for the CSPX4 sample with highest conductivity.

**Figure 6:**
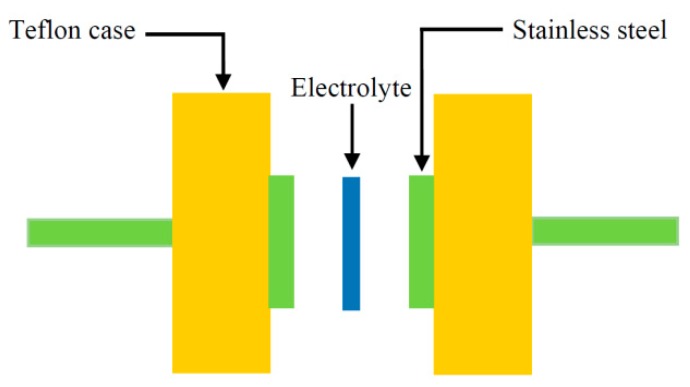
Schematic diagram for TNM and LSV measurements.

Along with TNM analysis, it is also of great importance to study linear sweep voltammetry (LSV) of the samples to determine the application suitability, in which the electrochemical stability of the samples can be investigated. From Figure 5, the LSV plot for the CSPX4 sample with highest conductivity is shown. The potential window, where the electrochemical stability is examined, is another crucial parameter that has to be evaluated before being used in the electrochemical devices (e.g., supercapacitors) [58]. In the recording LSV, two stainless steel electrodes were used to check the electrochemical stability of the polymer electrolyte as schematically presented in Figure 6. Within the potential window from 0 V to 3 V, a relatively large current is recorded at a certain potential at a sweep rate of 5 mV s^−1^. From an EDLC application perspective, the results revealed a satisfactory finding that the potential window of the blend is electrochemically stable within 2.43 V [59]. Monisha et al. [60] have shown a threshold potential during the current flowing through the cells. Shukur et al. [61] have documented a decomposition potential at 2.10 V for lithium salt-based biopolymer electrolyte. It is concluded that the obtained potential stability (potential window) of the relatively high conducting electrolyte in the present work is suitable for energy storage device applications. However, in our work, the increase in current beyond 2.43V was attributed to the electrolyte decomposition at the inert electrode surface [62]. In comparison to ammonium salt-based polymer electrolytes, the data results are quite close. Likewise, in another work, a plasticized system of chitosan-polyvinyl alcohol-NH_4_NO_3_ showed an electrochemical stability up to 1.70 V as documented by Kadir and Arof [63]. Noor and Isa [64], have also examined the cellulose-NH_4_SCN system, which was electrochemically stable up to 1.70 V.

#### 2.3.3. CV and Capacitance Study

**Figure 7:**
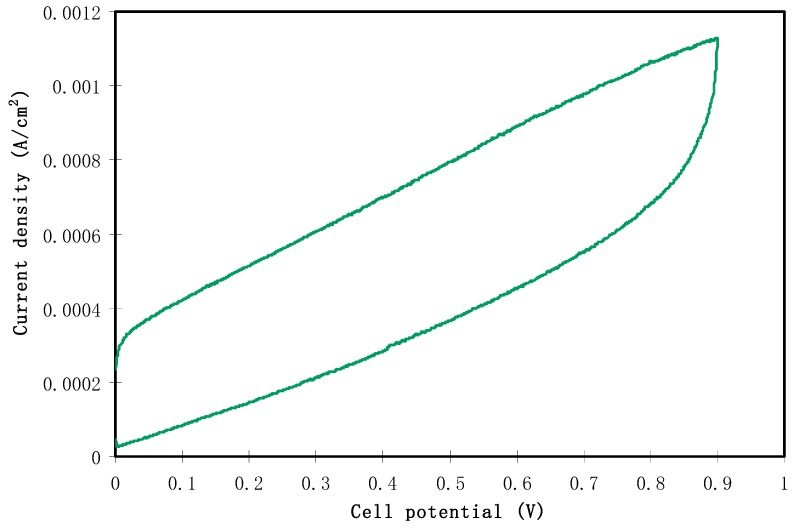
Cyclic voltammetric plot of the constructed EDLC in the potential range from 0 to 0.9 V at 50 mV s^−1.^

**Figure 8:**
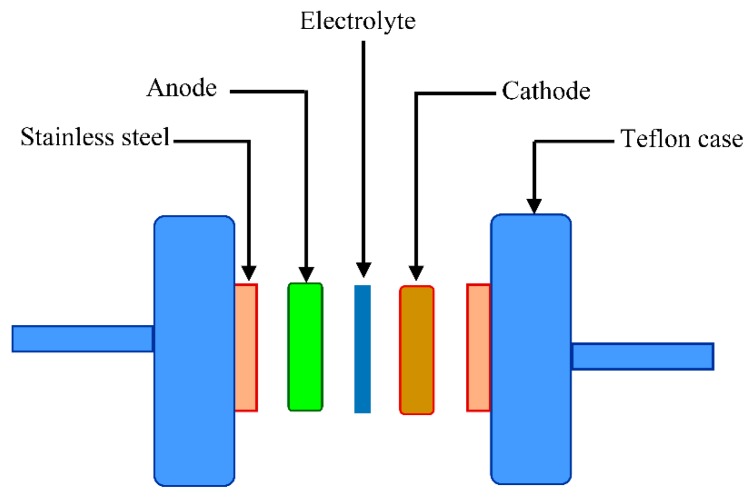
Schematic presentation of the fabricated electric double layer capacitor (EDLC) cell.

The fabricated EDLC is characterized in terms of a double layer using cyclic voltammetry (CV) at a sweep rate of 50 mV s^−1^, as shown in Figure 7. The characteristic feature of the fabricated EDLC is clearly seen in the CV profile which appears in a rectangular shape. The cyclic voltammetry can also observe the nature of charge storage at the interfaces between electrodes and electrolytes in EDLC studies. In other words, there is an insight into distinguishing between Faradaic and non-Faradaic processes from the CV [65]. From Figure 7, the performance of assembled cells at room temperature can be seen in the CV, indicating the absence of any oxidation-reduction reaction (redox) peak. This suggests the presence of a perfect capacitor without any contribution of redox process at the interfacial region [66]. Consequently, the rectangular shaped CV feature is considered as a strong evidence of a rapid current response to the applied voltage. This characteristic shape of our CV of the EDLC is quite similar to those reported in the literature [67,68,69]. The appearance of a non-rectangular shape is evidenced by two factors; firstly, fast build-up of the electric double layer, and secondly, internal resistance of EDLC. In addition to double layer formation, there is no hump feature in the CV, indicating the absence of any Faradaic reaction [70]. All these emphasize the EDLC formation at the interfacial region, using the accumulation mechanism of ion adsorption at the activated carbon [71]. Dealing with the mechanism of energy storage in microporous materials can be of great importance for both fundamental and industrial aspects. The great advance in designing supercapacitors with a relatively high performance enables researchers to develop this technology for industry applications in the near future. It is also considered as a breakthrough to some extent in the field of supercapacitor fabrication. Additionally, encouraging researchers to verify that the mechanism of charge storing—in terms of experimentation and theory—is necessary [72]. Eftekhari A. [73] has, however, shown a review where the energy storage mechanism is based on accommodation of ions within microporous and mesoporoes rather than the formation of a double layer capacitor. Accordingly, this mechanism is different to some extent from the classical model in which the ions facilitate the charge separation and storage in energy storage devices. On the other hand, the classical models are based on the inner layer which is formed due to the electrostatic forces; thereby the charged species are accumulated on the surface of the electrode without any real chemical interaction. Therefore, the double layer charging on the electrode surface should be ideally polarizable [74]. The schematic configuration of the EDLC cell is illustrated in Figure 8. The specific capacitance ($C_{s}$) of the EDLC can be calculated from the CV plot, which can be calculated using the following equation:

(4) $$C_{s}=\;\int_{v_{i}}^{v_{f}}\frac{I\left(V\right)dV}{2mv\;\left(V_{f}-V_{i}\right)}$$

where *V_f_* and *V_i_* are the final and initial voltage, respectively. *m* is the mass of active material, *v* is the scan rate and (V)dV is the area of the CV plot, which is obtained via OriginPro 8.5 software. The value of $C_{s}$, calculated for a scan rate of 50 mV s^−1^, is 3.80 F/g. This value is higher than other proton-based EDLC studies. Shukur [75] reported a range of $C_{s}$ from 1.14 to 3.64 F/g as the scan rate varied from 2 to 20 mV/s for a CS:starch:NH_4_Cl glycerol system. The author stated that the scan rate may affect the value of specific capacitance of the EDLC. Low scan rate causes the ions to properly conduct and form a charged double layer, and thus results in a higher capacitance value. An EDLC cell by Shuhaimi [76] with methylcellulose-NH_4_NO_3_ electrolyte obtained a $C_{s}$ value of 1.67 F/g. Liew et al. [77] reported a value of 0.14 F/g for PVA-NH_4_C_2_H_3_O_2_ based EDLC.

## 3. Materials and Methods

### 3.1. Materials and Sample Preparation

Chitosan (CS) as a natural biomolecule (average molecular weight 310,000–375,000g/mol) and poly (ethylene oxide) (PEO) powder (average molecular weight 300,000 g/mol) materials were purchased from Sigma-Aldrich (Sigma-Aldrich, Warrington, PA, USA). In this work, 1 g of CS was dissolved in 50 mL of 1% acetic acid for 90 min at room temperature, and separately 30 wt% (0.4285 g) of PEO was then dissolved in 50 mL of 1% acetic acid. Subsequently, the solutions were mixed together and stirred for 3h to obtain CS:PEO (70:30) polymer blends. Then, for the solution of CS:PEO, various amounts of NH4SCN, from 10 to 40 wt% in steps of 10 wt.%, were added separately under continuous stirring to fabricate CS:PEO: NH_4_SCN electrolytes. The final solutions were coded as CSPX0, CSPX1, CSPX2, CSPX3, and CSPX4 for CS:PEO incorporated with 0, 10, 20, 30, and 40 wt% of NH4SCN, respectively. The obtained electrolytes were put in the labeled Petri dishes and left at room temperature in a desiccator to obtain dried films. By this, the obtained films were free of solvent and the resulted polymers were utilized as raw materials. The thickness of the electrolyte samples werein the ranges 123–125 μm.

### 3.2. Structural and Impedance Characterizations

Fourier transforms infrared (FTIR, Thermo Scientific, Nicolet iS10,Perkin Elmer, Waltham, MA, USA) spectroscopy measurements were carried out via a Spotlight 400 Perkin-Elmer system with a resolution of 1 cm^−1^ (450–4000 cm^−1^). The electrical impedance properties of the prepared SPE films were analyzed at room temperature, using a HIOKI 3532–50 LCR HiTESTER controlled by a computer via software over the frequency range of 42 Hz to 5000 kHz. From the computer, the measurements as well as the analysis of both the imaginary and real parts of the impedance spectra could be performed. Discs with a diameter of 20 mm were prepared from the SPE films, and then sandwiched between a pair of stainless steel electrodes under spring pressure.

### 3.3. TNM and LSV Measurements

V&A Instrument (Neware, Shenzhen, China) DP3003 digital DC power supply was employed to perform the transference number (TNM) analysis using DC polarization technique.Prior to the measurement, the high conducting electrolyte samples were sandwiched between blocking stainless steel electrodes in a Teflon holder. The polarization was carried out at 0.8 V and the DC current was monitored as a function of time at the room temperature. The potential window, i.e., stable potential, of the electrolyte was also recorded using linear sweep voltammetry (LSV) analysis (DY2300 potentiostat, Neware, Shenzhen, China) at a sweep rate of 5 mV/s.

### 3.4. EDLC Preparation

The electrode for EDLC study was fabricated using polyvinylidene fluoride (PVdF), activated carbon and carbon black materials. Under moderate stirring, 0.50 g of PVdF was added into 15 mL N-methyl pyrrolidone (NMP). Activated carbon and carbon black powder of 3.25 g and of 0.25 g, respectively, were mixed using planetary ball miller to gain a homogeneous solution. This dispersed solution was then poured into the PVdF solution. The mixture was then cast on an aluminum foil using a doctor blade technique and heated at 60 °C for a certain time. The electrodes were placed in a desiccators containing silica gel to obtain acceptable dryness state. As usual, the resulting relatively high conducting electrodes were sandwiched between two carbon electrodes and packed in CR2032 coin cells. The thickness of the electrodes was optimized to 0.0025 cm.Digi-IVY DY2300 Potentiostat was employed to perform cyclic voltammetry (CV) of the EDLC system at the potential window of 0 to 0.9 V and at a sweep rate of 50 mV/s.

## 4. Conclusions

In conclusion, we have shown the suitability of the various properties of polymer blend electrolytes of chitosan (CS):poly (ethylene oxide) (PEO):NH_4_SCN systems for energy storage devices. The complex formation via the FTIR study was confirmed by observing the shift and the decrease in intensity of FTIR bands corresponding to functional groups. The EIS analysis showed the existence of bulk resistance in which the semicircle diameter shrunk with increasing salt concentration. The calculated maximum DC conductivity value was found to be 2.11 × 10^−4^ S/cm. The preliminary results from CV, LSV, and EIS techniques revealed the mechanism of storage is double layer charging. The electrochemical stability of the system exceeded the aqueous counterpart and a relatively high DC conductivity was obtained for CS:PEO incorporated with 40 wt% of NH_4_SCN salt, respectively. The rectangular response in the CV, a high ionic nature of charge transfer, and a relatively high electrochemical stability provide strong evidence for applying these systems as capacitors. The value of $C_{s}$ for the fabricated EDLC at a scan rate of 50 mV s^−1^ was found to be 3.80 F/g.

## Figures and Tables

**Table 1:** Calculated DC conductivity for neat CS:PEO and blend electrolyte films at room temperature.

Sample Designation	DC Dconductivity (S cm^−1^)
CSPX 0	2.09 × 10^−10^
CSPX 1	7.69 × 10^−8^
CSPX 2	5.77 × 10^−7^
CSPX 3	1.49 × 10^−4^
CSPX 4	2.11 × 10^−4^
